# Multi-Head Attention-Based Long Short-Term Memory for Depression Detection From Speech

**DOI:** 10.3389/fnbot.2021.684037

**Published:** 2021-08-26

**Authors:** Yan Zhao, Zhenlin Liang, Jing Du, Li Zhang, Chengyu Liu, Li Zhao

**Affiliations:** ^1^Key Laboratory of Underwater Acoustic Signal Processing of Ministry of Education, Southeast University, Nanjing, China; ^2^Computational Intelligence Group, Northumbria University, Newcastle upon Tyne, United Kingdom; ^3^National Subsea Centre, Robert Gordon University, Aberdeen, United Kingdom; ^4^School of Instrument Science and Engineering, Southeast University, Nanjing, China

**Keywords:** depression, LSTM, multi-head attention, frame-level feature, deep learning

## Abstract

Depression is a mental disorder that threatens the health and normal life of people. Hence, it is essential to provide an effective way to detect depression. However, research on depression detection mainly focuses on utilizing different parallel features from audio, video, and text for performance enhancement regardless of making full usage of the inherent information from speech. To focus on more emotionally salient regions of depression speech, in this research, we propose a multi-head time-dimension attention-based long short-term memory (LSTM) model. We first extract frame-level features to store the original temporal relationship of a speech sequence and then analyze their difference between speeches of depression and those of health status. Then, we study the performance of various features and use a modified feature set as the input of the LSTM layer. Instead of using the output of the traditional LSTM, multi-head time-dimension attention is employed to obtain more key time information related to depression detection by projecting the output into different subspaces. The experimental results show the proposed model leads to improvements of 2.3 and 10.3% over the LSTM model on the Distress Analysis Interview Corpus-Wizard of Oz (DAIC-WOZ) and the Multi-modal Open Dataset for Mental-disorder Analysis (MODMA) corpus, respectively.

## 1. Introduction

Depression is a prevalent mental disorder, affecting millions of human beings all over the world (Organization, 2017). Depression not only makes patients bear psychological pain, pessimism and, self-accusation but also leads to a high possibility of disability and death (Hawton et al., 2013). It can bring a severe burden on individuals and families. Moreover, the particularity of mental disorders makes them difficult to diagnose. Most people with depression do not seek medical advice or even ignore it. Its diagnosis mainly relies on the self-report of patient or explicit severe mental disorder symptoms (Hamilton, 1960; Zung, 1965). There are also other evaluations, such as the 9–item Patient Health Questionnaire (PHQ–9) (Kroenke and Spitzer, 2002), the PHQ–8 (Kroenke et al., 2009), and so on. Influenced by subjective factors, such methods have some limitations. Therefore, providing an effective and objective method, as an auxiliary standard, for detecting depression, is of vital significance.

In recent years, myriad models have been proposed for automatic depression detection. Senoussaoui et al. (2014) showed that an i-vector-based representation of short-term acoustic features, which contains 20 static Mel Frequency Cepstral Coefficients (MFCC) and 40 dynamic MFCC coefficients, is effective for depression classification based on different regression models. Yang et al. (2017) proposed a Deep Convolutional Neural Network (DCNN) with the text, video, and audio descriptors for detecting depression. Rodrigues Makiuchi et al. (2019) proposed a multimodal fusion of speech and linguistic representations for depression detection. By parallel employing the textual, audio, and visual models, the acquired features compose the input features of the full connection layer. Jan et al. (2017) proposed a Convolutional Neural Network (CNN) architecture for automatic depression prediction. Various frame-level features were extracted to obtain distinctive expression information. Yin et al. (2019) proposed a Hierarchical Bidirectional LSTM with text, video, and audio features for depression prediction. Li et al. (2019a) employed CNN for mild depression recognition based on electroencephalography. We observe that most of the proposed models (Senoussaoui et al., 2014; Jan et al., 2017; Yang et al., 2017; Rodrigues Makiuchi et al., 2019; Yin et al., 2019) rely on multimodal calculation, instead of focusing on the internal relation of the speech signal. We believe that making full use of the emotional information at all times is the key to provide an effective model for depression classification.

Therefore, to emphasize the key information of speech signals, an improved attention-based LSTM model is proposed for automatic depression detection in this research. First, we apply frame-level features for LSTM. The frame-level features keep the inherent emotional information of the speech sequences. Moreover, its variable length is suitable for LSTM. Second, we apply multi-head time-dimension attention for LSTM output to utilize the critical inherent information. Besides, the multi-head attention helps linearly project the LSTM output into different subspaces for various context vectors with reduced dimensions. To indicate the model efficiency, we evaluate the proposed model on the DAIC-WOZ and MODMA corpora.

The rest of the study is organized as follows. Section 2 describes related studies. Section 3 Analysis introduces the frame-level features and the selection. The proposed attention-based LSTM model is introduced in section 4. The databases and experiment results are provided in section 5. Section 6 discusses the experiment results. Section 7 concludes this study.

## 2. Related Work

### 2.1. Deep Learning Models

For depression detecting, the machine learning algorithms were initially utilized, such as support vector machine (SVM) (Long et al., 2017; Jiang et al., 2018) and Gaussian mixture model (GMM) (Jiang et al., 2018). In recent years, deep neural networks have been widely used for detecting depression (Jan et al., 2017; Yang et al., 2017; Li et al., 2019a; Rodrigues Makiuchi et al., 2019; Yin et al., 2019). Previous studies such as Yang et al. (2017) and Jan et al. (2017) employed CNN as the classification model with multiple features for depression prediction. Making full use of the multimodality features is the key success of their models. Yin et al. (2019) used a Hierarchical Bidirectional LSTM network for the processed sequence information to predict depression. Besides utilizing multimodality features, their work focused on extracting time sequence information to inform prediction. Various methods are developed for the classification of speech emotions (Tiwari et al., 2020; Abbaschian et al., 2021). In addition, studies by (Li et al., 2019b; Xie et al., 2019; Zhao et al., 2019) has proved that the LSTM network is effective for processing sequential signals. Since the existing studies lack exploring the inherent relationships of the speech signals, we proposed a multi-head time-dimension attention LSTM model for depression classification. The proposed method is utilized for emphasizing the information of emotional salient regions to boost the classification performance for depression detection.

### 2.2. Attention Mechanism

Recently, the attention mechanism has achieved great success in computer vision. Xiao et al. (2015) applied visual attention to deep neural network for fine-grained classification tasks. Zhao et al. (2017) proposed a diversified visual attention network for object classification. The core idea is that the attention of a person depicts different priorities for various parts of an image. Inspired by such a strategy, the attention mechanism is introduced into speech emotion recognition. Mirsamadi et al. (2017) proposed local attention using recurrent neural networks for speech emotion recognition. Xie et al. (2019) used both time and feature dimension attention mechanism to achieve better performance for speech emotion recognition. Li et al. (2019b) explored the effectiveness of the self-attention mechanisms and multitask learning for speech emotion recognition. Specifically, previous studies by Mirsamadi et al. (2017) and Xie et al. (2019) have mainly focused on calculating different attention weightings for different parts of speech waveforms.

With the widely use of attention mechanism, a multi-head attention scheme has been proposed Vaswani et al. (2017) and introduced to many areas. Jiang et al. (2019) used Bidirectional Encoder Representations from Transformers (BERT) as the encoder for unsupervised pre training. Lian et al. (2019) proposed a multi-head attention framework, fusing the context, the emotional information of speech and speakers, to reach better performance for speech emotion classification. The earlier literatures (Mirsamadi et al., 2017; Lian et al., 2019; Li et al., 2019b; Xie et al., 2019; Abbaschian et al., 2021) indicate that the attention mechanism is effective for mining the inherent emotional information from speech. Hence, it is suitable for the study to apply such an attention mechanism for depression speech detection.

## 3. Acoustic Features Analysis

The depression prediction with respect to speech comprises speech processing and classification methods based on the extracted features. The performance rate of a classifier largely relies on the type of extracted features. Many hand–crafted features have been discovered and used for improving prediction performances. These include prosodic features (Yang et al., 2017), spectral features (Senoussaoui et al., 2014; Yang et al., 2017; Rodrigues Makiuchi et al., 2019; Yin et al., 2019), and energy related features (Yang et al., 2017), e.g., Previous studies indicate that speech emotions have an inherent relationship with depression detection. In this study, we evaluate the widely used ComParE openSMILE features (Schuller et al., 2016; Jassim et al., 2017) and adopt some speech features as acoustic descriptors for depression detection. Table 1 describes the frame-level speech features.

**Table 1:** Frame-level speech features.

**Acoustic features**	**Description**
F0	Pitch frequency
Jitter	The average absolute difference between the
	consecutive periods
Shimmer	The average absolute difference between the
	interpolated peak amplitudes of consecutive periods
Loudness	The loudness and delta regression of loudness
MFCC	MFCC and delta regression of MFCC
Pcm_Mag	Mel spectral
Lpc	Linear predictive coding coefficients
LspFreq	Line spectral pair frequency
voiceProb	The voicing probability
harmonicERMS	Harmonic component root mean square energy
noiseERMS	Noise component root mean square energy
HNR	Log harmonics-to-noise ratio

**Figure 1:**
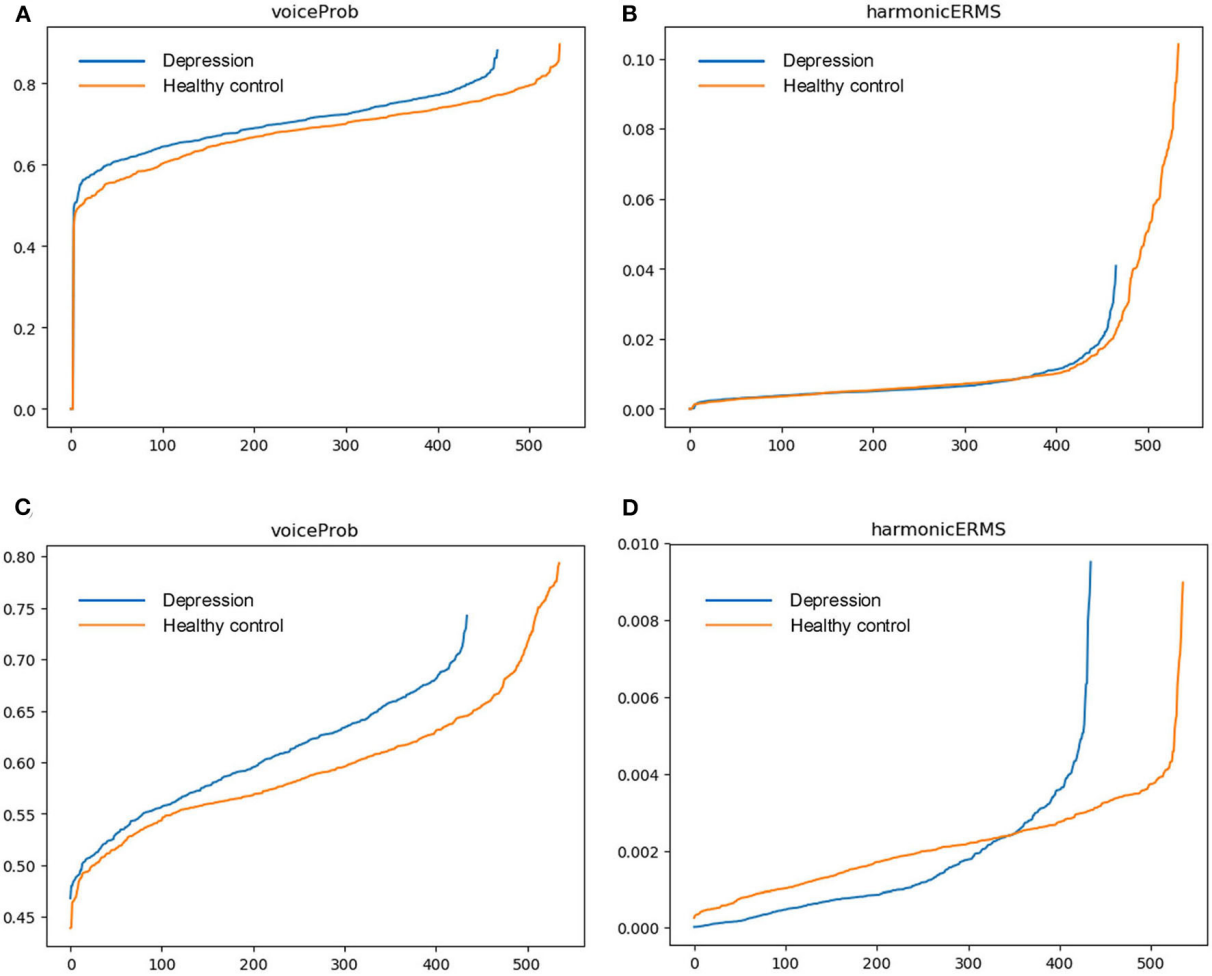
Feature (voiceProb and harmonticERMS) comparison. **(A,B)** are features mean values on the Distress Analysis Interview Corpus - Wizard of Oz (DAIC-WOZ) corpus, while **(C,D)** are features mean values on the MODMA corpus.

**Figure 2:**
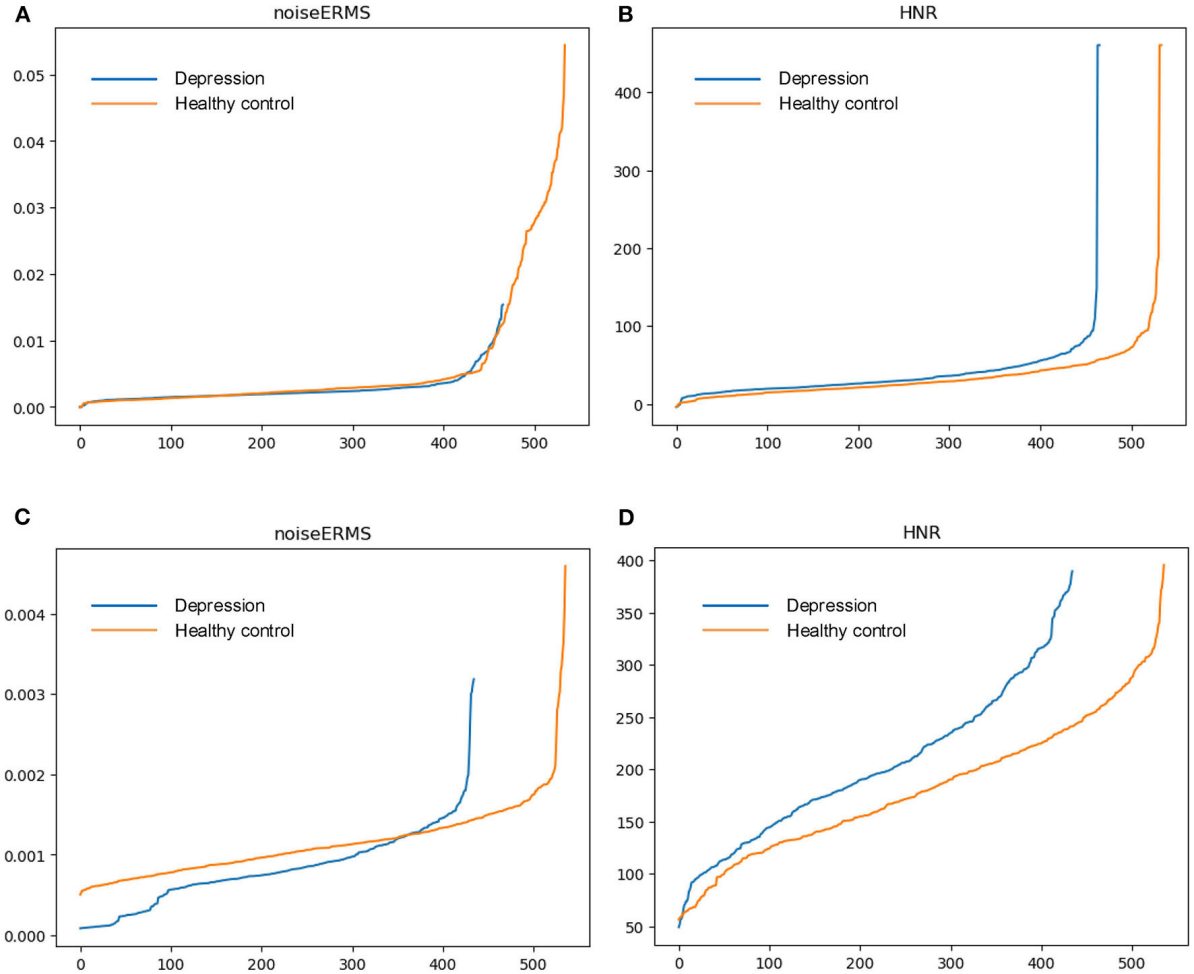
Feature (noiseERMS and HNR) comparison. **(A,B)** are feature mean values on the DAIC-WOZ corpus, while **(C,D)** are feature mean values on the MODMA corpus.

To evaluate and visualize the impact of features on detection, samples from DAIC-WOZ and MODMA corpora are taken for comparison. For each feature, we calculate the mean value of speech segments and sort them in ascending order. Outliers cause an excessive gradient. To identify the effectiveness of features for prediction, we take speech samples from DAIC-WOZ and MODMA corpora and calculate the mean value of the features over timeframes. Figures 1, 2 exhibit the mean values of four features. The x-axis represents the sample numbers and the y-axis represents the amplitude. They show that the HNR feature has the largest distinction among the four features. For voiceProb, it has many overlaps for samples on DAIC-WOZ corpus, which means that it may not be effective for depression as a single feature. The same situation is observed on harmonticERMS and noiseERMS on the DAIC-WOZ database.

**Figure 3:**
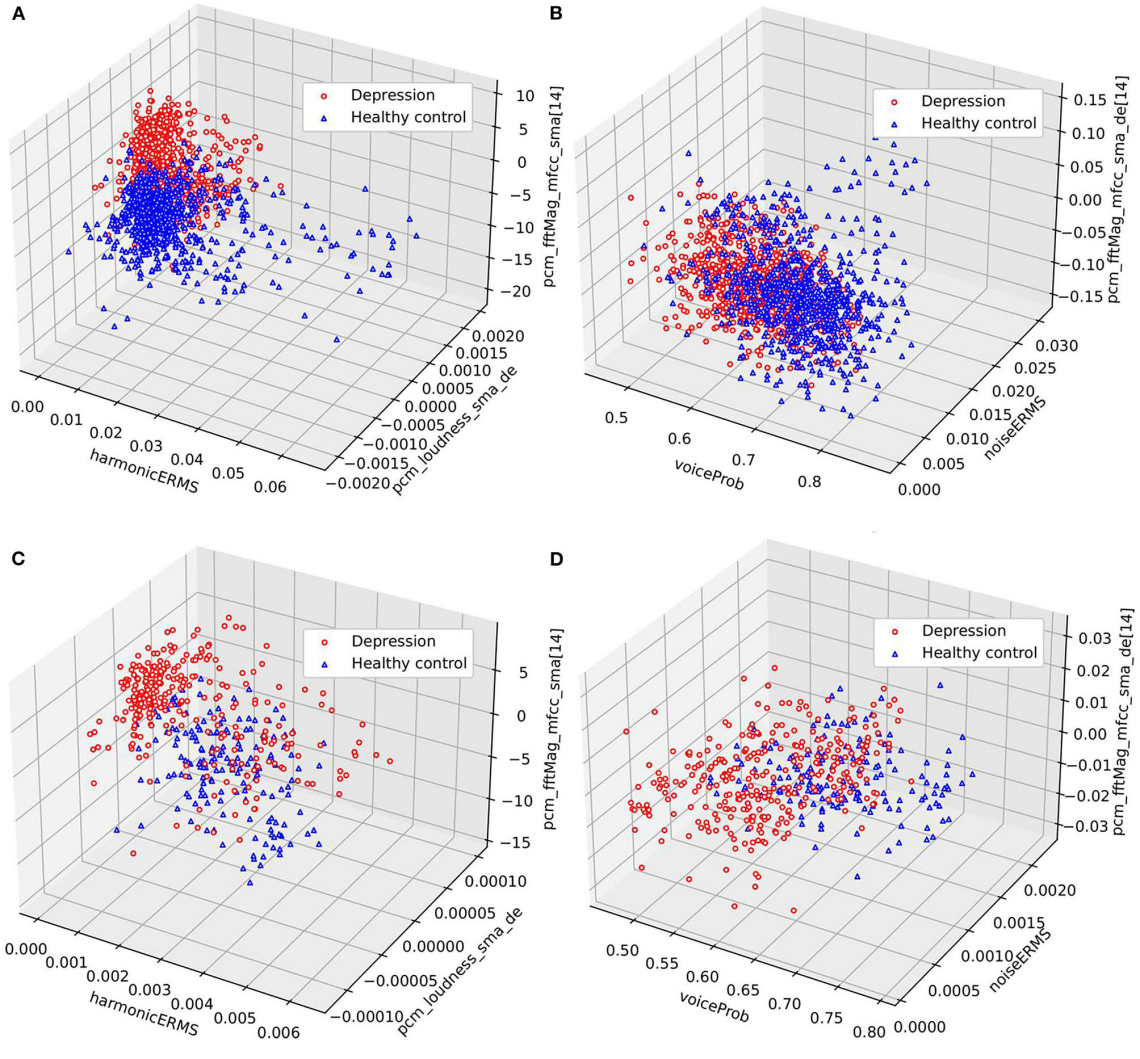
Cluster comparisons. **(A,B)** are clustering results on the DAIC-WOZ corpus, while **(C,D)** are clustering results on the MODMA corpus.

Furthermore, we conduct cluster analysis on DAIC-WOZ and MODMA corpora respectively. The mean values of the features over timeframes are calculated as before. The distributions of samples under different feature combinations are shown in Figure 3. The cluster results reveal the differences between the depression and normal samples. In Figures 3A,C, most of the depression samples tend to be lower on harmonicERMS and higher on MFCC, while the distributions of the two types of samples are roughly the same in terms of pcm_loudness_sma_de, which is consistent with the previous results. The second combination is voiceProb, noiseERMS, and the delta regression of MFCC. According to the previous analysis, there is significant overlap on voiceProb and noiseERMS on the DAIC-WOZ corpus. However, it can be seen from Figures 3B,D that there are also two distinct cluster centers despite more overlapping parts compared to Figures 3A,C both on DAIC-WOZ and MODMA corpora. This phenomenon indicates that a combination of two or more features can improve the ability to distinguish depression. It also demonstrates the effectiveness of the frame-level features in the identification of depression. Finding an effective model to expand the gap between depression and normal samples is right way to go.

## 4. Multi-Head Attention-Based LSTM

The attention mechanism has been introduced to many areas successfully (Xiao et al., 2015; Mirsamadi et al., 2017; Vaswani et al., 2017; Zhao et al., 2017; Jiang et al., 2019; Lian et al., 2019; Xie et al., 2019). The main idea of the attention mechanism is to pay more attention to a certain weight distinction. In the previous study, Xie et al. (2019) studied the effectiveness of frame-level speech features, which include temporal information as well as feature-level information. The final representations multiplied by the attention layer helps model to improve the performance. In this study, for mining the multiple representations with more emotional information, we introduce the multi-head attention mechanism to depression detection and further develop the attention-based LSTM model.

### 4.1. LSTM Model

Hochreiter and Schmidhuber (1997) first proposed LSTM. Gers et al. (2000) added the forgetting gate for LSTM and proved its effectiveness. In an LSTM cell, the forgetting gate is used for discarding the useless information of the previous moment and updating the cell state. The previously hidden layer output and the current moment input are used in the updating algorithm. Multiple structures have been proposed for improving the LSTM performance, e.g., the forgetting gate (Gers et al., 2000) and peepholes (Gers and Schmidhuber, 2000). In the previous work, Xie et al. (2019) proposed an attention gate for LSTM to reduce the number of training calculations. The experiments indicate that the attention gate can help improve the effectiveness of LSTM model training. Hence, in the study, we use the modified LSTM (Xie et al., 2019) as the baseline.

### 4.2. Multi-Head Attention

Vaswani et al. (2017) first proposed the multi-head attention scheme. By taking an attention layer as a function, which maps a query and a set of key-value pairs to the output, their study found that it is beneficial to employ multi-head attention for the queries, values, and keys. By linearly projecting the context vectors into different subspaces, the multi-head attention layer computes the hidden information, which shows better performance than that of single-head attention. Inspired by Vaswani et al. (2017), we calculate the output by weighted values, which are computed by queries and the corresponding keys.

Xie et al. (2019) has presented the time-dimension calculation for attention weighting:

(1) $$\begin{array}{l} s_{t}=softmax\left(o_{last}\times {\left(o_{all}\times W_{t}\right)}^{H}\right),o_{last}\in R^{B,1,Z} \end{array}$$

(2) $$\begin{array}{l} o_{t}=s_{t}\times o_{all},o_{all}\in R^{B,T,Z},s_{t}\in R^{B,1,T} \end{array}$$

where *s*_*t*_ donates the attention score of the time dimension, *o*_*last*_ represents the last time output and *o*_*all*_ is the all-time output. *B* represents the batch size, and *T* represents the number of time steps, while *Z* represents the feature dimension. The parameter 1 represents the last time step. *H* represents the transpose operator, and *W*_*t*_ represents the parameter matrix, while *o*_*t*_ donates the output of the time-dimension attention layer.

Formulas 1 and 2 are the single-head attention calculation. We only use two types of LSTM output for attention. The output of all time is essential because it contains all LSTM output information. The reason to choose the last time step output is that it includes the most redundant information among all time steps. For multi-head time-dimension attention computing, we also choose the two types of output to calculate the queries, keys, and values:

(3) $$\begin{array}{l} K_{i}=W_{i,k}\times o_{all}+b_{i,k},K_{i}\in R^{B,T,\frac{Z}{n}},W_{i,k}\in R^{Z,\frac{Z}{n}},b_{i,k}\in R^{\frac{Z}{n}} \end{array}$$

(4) $$\begin{array}{l} V_{i}=W_{i,v}\times o_{all}+b_{i,v},V_{i}\in R^{B,T,\frac{Z}{n}},W_{i,v}\in R^{Z,\frac{Z}{n}},b_{i,v}\in R^{\frac{Z}{n}} \end{array}$$

(5) $$\begin{array}{l} Q_{i}=W_{i,q}\times o_{last}+b_{i,q},Q_{i}\in R^{B,1\frac{Z}{n}},W_{i,q}\in R^{Z,\frac{Z}{n}},b_{i,q}\in R^{\frac{Z}{n}} \end{array}$$

where *K*,*V*,*Q* donate the value, key, and query. *n* is the number of attention heads and *b* means bias.

**Figure 4:**
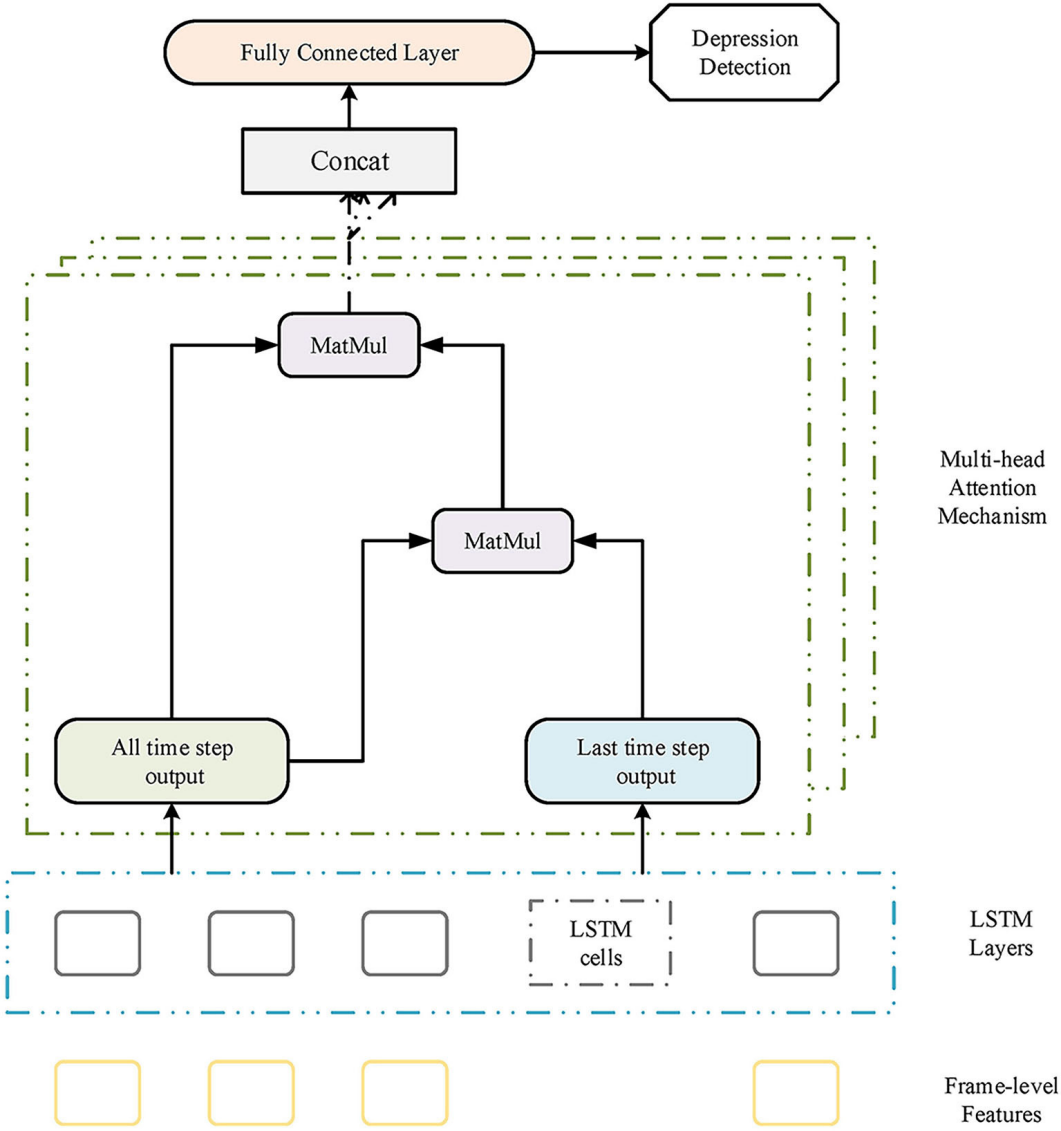
The structure of the proposed multi-head attention-based long short-term memory (LSTM) model.

The multi-head attention scores and context vectors are calculated as follows:

(6) $$\begin{array}{l} s_{i}=softmax\left(Q_{i}\times K_{i}^{H}\right),s_{i}\in R^{B,1,T} \end{array}$$

(7) $$\begin{array}{l} context_{i}=s_{i}\times V_{i},context_{i}\in R^{B,1,\frac{Z}{n}} \end{array}$$

(8) $$\begin{array}{l} CV=Concat\left(\left[context_{1},\ldots ,context_{n}\right]\right),CV\in R^{B,1,Z} \end{array}$$

where *s*_*i*_ represents the multi-head time-dimension attention score and *context*_*i*_ represents the reduced-dimension context vectors from each subspace. The overall structure of multi-head time-dimension attention is described in Figure 4. Next, the context vector is put into the full connection layer. The output is then sent to the softmax layer for final prediction.

## 5. Experiment and Results

### 5.1. Datasets

In this research, we evaluate the proposed model on DAIC-WOZ (Gratch et al., 2014) and MODMA (Cai et al., 2020) corpora. The DAIC data corpus contains clinical interviews designed to support the diagnosis of psychological distress conditions. The sampling rate is 16,000 Hz. The numbers of depression and healthy control samples randomly selected are 42 and 47, respectively. Then, we divide them into segments, which makes feature extraction more convenient. We obtain 2,156 depression segments and 2,245 healthy control segments from the selected samples. To ensure the effectiveness of the fragments, abnormal segments, which are <3 s with litter information or larger than 20 s, are discarded in this research. Finally, we utilize 3,401 and 1,000 audio segments, which are randomly sorted by the software, as the train set and the test set, respectively.

The database contains 52 samples on the MODMA database, with 23 depression and 29 healthy control samples. We also divide them into sentences. Compared with samples in the DAIC-WOZ corpus, samples of MODMA contains much more information with an average duration of over 10 s. We also discard the abnormal segments, which are much larger than other segments. At last, we several 1,321 segments. We randomly split them into two different sets (train set and test set). The train set includes 971 segments while the test set contains 350 segments. Both of the corpora are grouped into two categories (depression and healthy control).

### 5.2. Multi-Head Time-Dimension LSTM

We utilize the attention mechanism to capture the key information from the depression speech. In the previous study, Xie et al. (2019) used single-head attention for emphasizing the reverent key information related to the task. In this study, we proposed multi-head time-dimension attention for depression detection. To prove its validity, we conduct experiments for comparison with LSTM models. We use three types of LSTM models and evaluate them on DAIC-WOZ and MODMA corpora. The models are: (1) LSTM. (2) LSTM+T, which is time-dimension attention LSTM (Xie et al., 2019). (3) LSTM+*n*T, which is the proposed multi-head time-dimension LSTM, and *n* represents the head number. The proposed models, including the LSTM and multi-head time-dimension-based LSTM, are composed of two LSTM layers. The number of hidden units for the first LSTM layer is 512 and that of the second LSTM layer is 256. The size of the fully connected layer is [128, 12]. The learning rate is set as 0.0001, and the batch size is 64. We extract the acoustic features mentioned above by openSMILE Eyben et al. (2010) and use them as the input of the proposed model. Instead of pretraining on other databases, we train the models directly on DAIC-WOZ and MODMA corpora. Table 2 shows the experimental results.

**Table 2:** Unweighted average recalls (UARs) of different models on DAIC-WOZ and MODMA corpora.

**Model**	**UAR**	
	**DAIC-WOZ(%)**	**MODMA(%)**
LSTM	91.2	88.6
LSTM+T	92.1	96.6
LSTM+2T	92.9	**98.9**
LSTM+4T	**93.5**	98.3
LSTM+8T	92.5	98.0

*Bold values represent the best results in the comparison*.

As described in Table 2, the LSTM+T model has better results than those of the LSTM model, while LSTM+nT models acquire the best performance on both DAIC-WOZ and MODMA corpora. We choose unweighted average recall (UAR) to evaluate the effectiveness of the two feature sets for different databases. UAR is defined as: UAR=$\frac{1}{N}\overset{N}{\underset{i=1}{\sum }}\frac{c_{i}}{n_{i}}$, where *c*_*i*_ represents the correctly classified sample number of *i* category, *n*_*i*_ represents sample number of *i* category and *N* represents categories. The time-dimension attention shows its reliability for depression detection, by improving 0.9 and 8.0% on DAIC-WOZ and MODMA corpora, respectively. The LSTM+*n*T models achieve the best UARs (93.5% on DAIC-WOZ and 98.9% on MODMA) in the experiments. The model UAR is 93.5% on DAIC-WOZ with 4-head and it is up to 98.9% on MODMA with 2-head.

**Figure 5:**
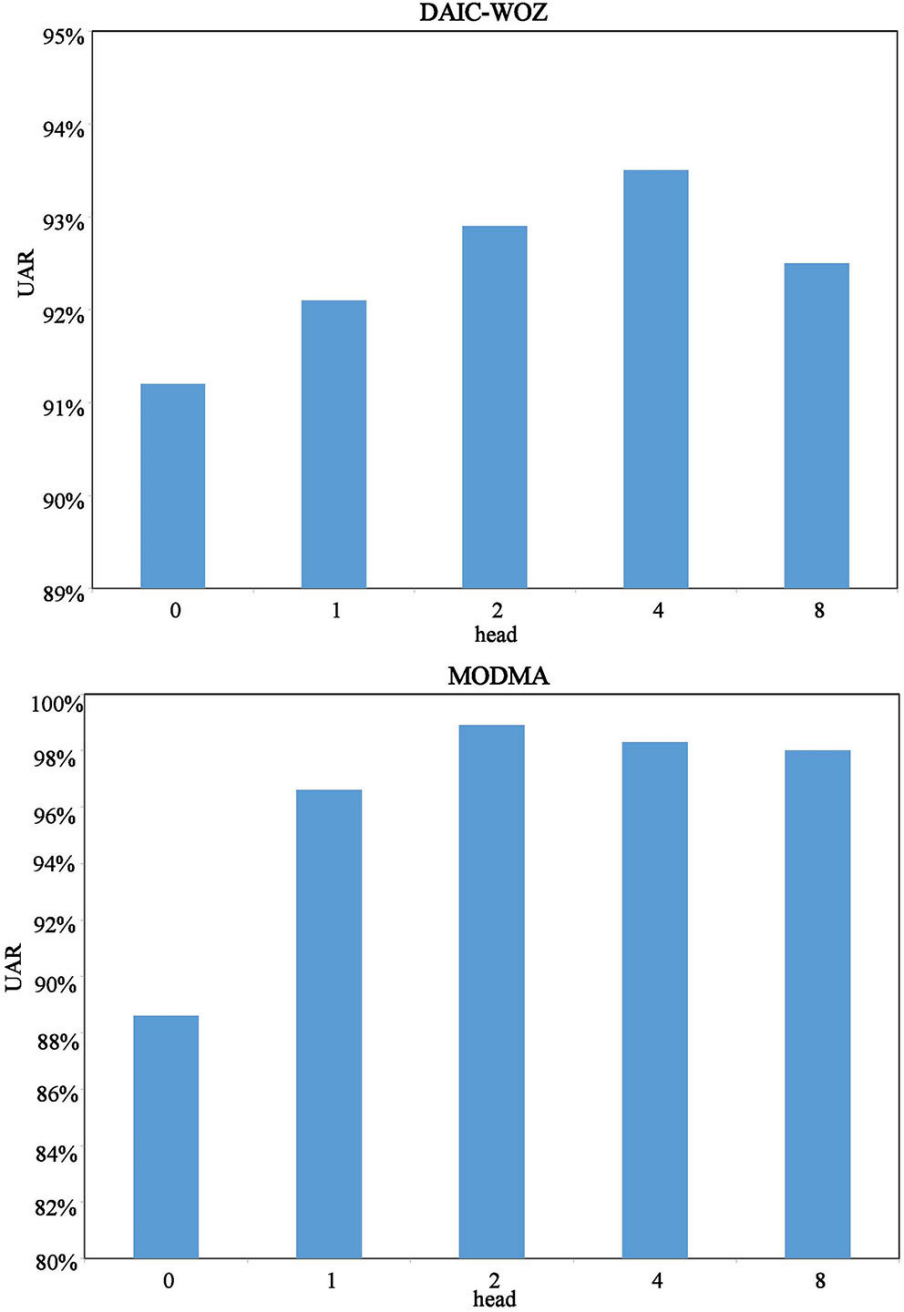
Result tendency of different models on the DAIC-WOZ and MODMA corpora.

Figure 5 shows the tendency of the models. Zero-head means LSTM while 1-head denotes the LSTM+T model. For experiments on DAIC-WOZ, we observe that the multi-head time-dimension attention models tend increasing first and decreasing subsequently. We believe it is normal for multi-head attention calculation to illustrate such a performance behavior. The reason is that the increase of head number cannot always help the model obtain better performance. There must be a boundary for it. Since the tendency proves the boundary, we believe the multi-head time-dimension attention LSTM has achieved the best UAR with 4-head. For the MODMA corpus, we can see that attention is effective. All models with attention have a high UAR of over 95%. The phenomenon could be caused by distinguishing features on the MODMA corpus, which can be proved on the feature comparison of the frame-level speech feature section. The 2-head time dimension achieves the best result of 98.9%. If we put the single-head attention into consideration, it still tends increasing first and then decreasing. The experiment results prove the effectiveness of the proposed multi-head time-dimension attention.

**Figure 6:**
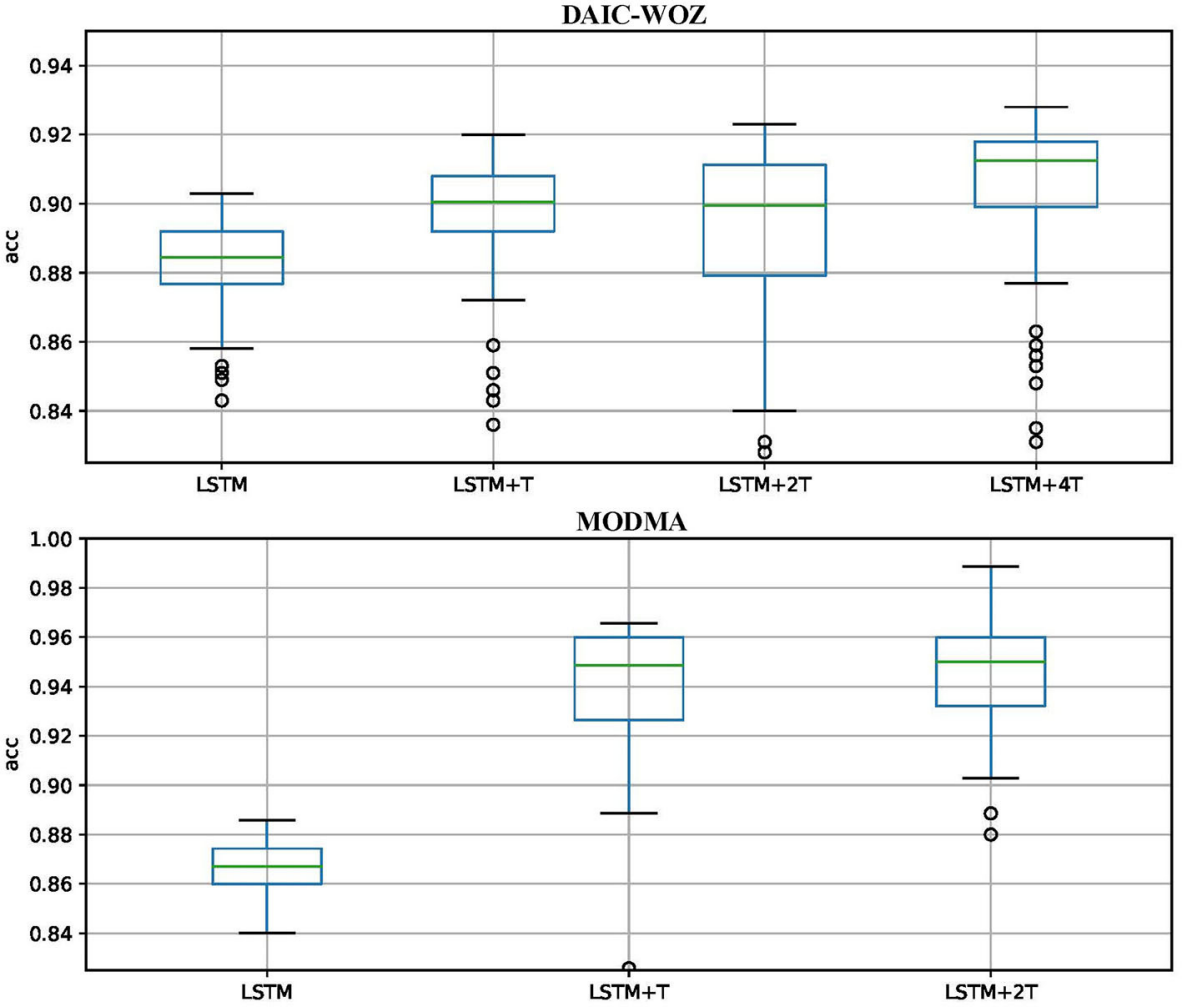
Stability of models on test sets.

Figure 6 shows the stability of models on the test set. The y-axis represents accuracy (UAR), and the x-axis represents the models. We exhibit the results from LSTM to the best model on the DAIC-WOZ and MODMA corpora. The blue rectangular box height indicates the stability of the model, and the lines inside the box are the stable UAR. On the test set, we could obtain thousands of results when the model converges. The stability in this study means most test results are inside the box range. The stable UAR means the median of results. The two lines outside the box mean the highest and lowest UARs. Circles represent outliers. As shown in the figures, the LSTM+*n*T model achieves higher stable UAR than those of LSTM and LSTM+T models on both test corpora. The overall performance indicates the LSTM+*n*T models are more reliable than other models.

**Figure 7:**
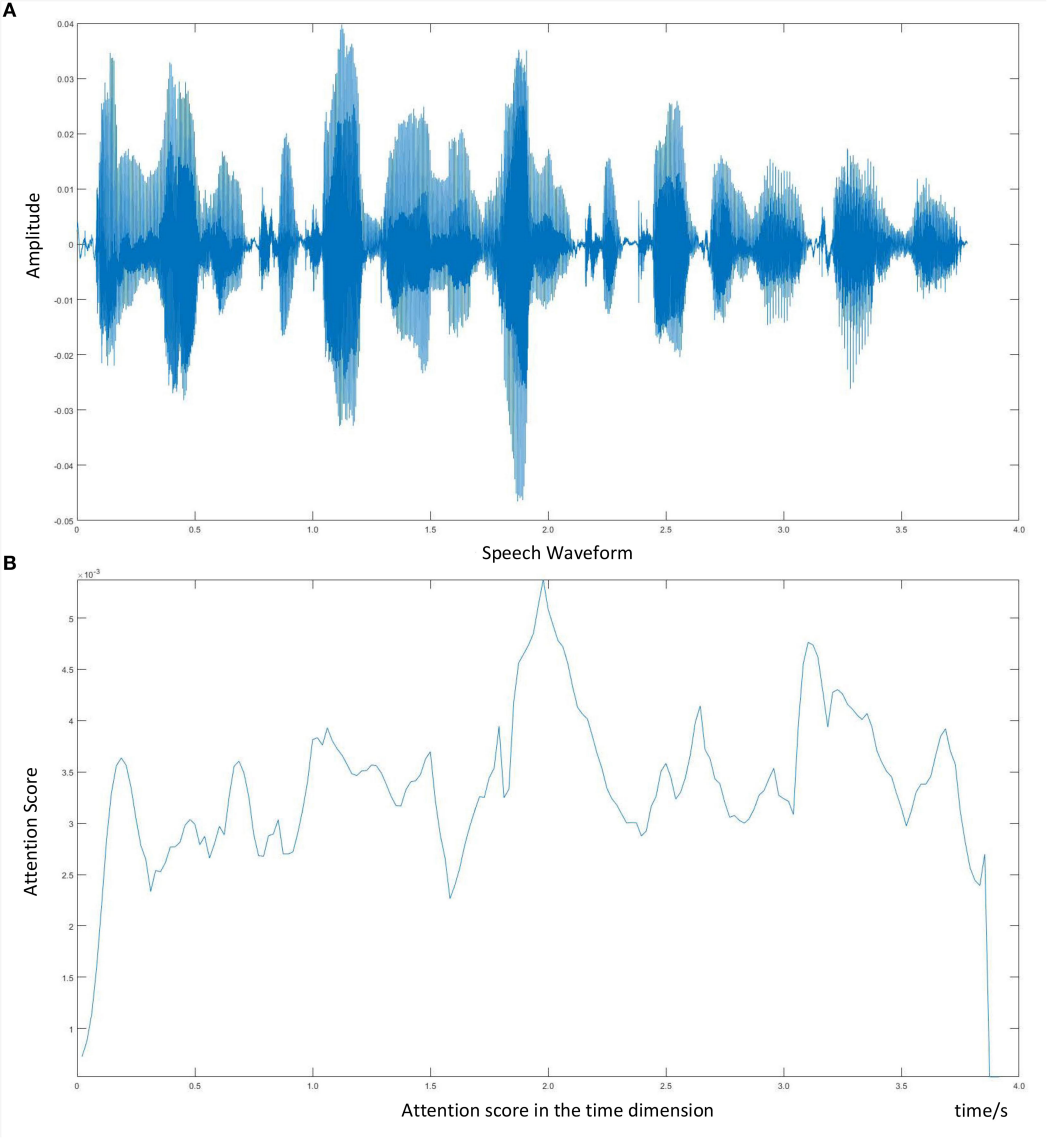
Visualization of speech waveform and corresponding attention score. **(A)** Speech waveform. **(B)** Attention score in the time dimension.

For a better understanding of the depression patterns in speech signals, we draw the speech waveform as well as its corresponding attention score, which is shown in Figure 7. We experiment on one audio clip, using the 4-head attention LSTM model, and visualize one of the attention scores. What can be seen from Figure 7 is that the multi-head attention mechanism endows diverse weights for the salient regions. For example, the attention score changes with the fluctuation of the audio clip and achieves a peak around 2 s. Moreover, with the amplification of the emotional part, we can pay more attention to the negative regions, which is beneficial for depression detection from speech.

## 6. Discussion

In this study, we extract frame-level features to detect depression. In the previous study, Long et al. (2017) and Jiang et al. (2018) studied the speech features using different classifiers. The developments of Long et al. (2017) and Jiang et al. (2018) prove the effectiveness of MFCC, loudness, and F0 features. Therefore, we adopt those widely used features as parts of this study. To evaluate the effectiveness of features, we conduct a comparison between depression and normal samples to visualize the impact of features on detection. The results indicate that enhancing the emotional region of speech is a fundamental part of better depression classification.

Table 2 exhibits the results of LSTM and multi-head time-dimension. We could easily find that LSTM obtains the worst results on both DAIC-WOZ and MODMA corpora. The best LSTM+nT model improves by 2.3 and 10.3% on DAIC-WOZ and MODMA, respectively. It indicates that the multi-head attention mechanism helps the model to emphasize the key time information of sequence. Besides that, we find that the best results of the multi-head time-dimension attention-based LSTM model achieve the 1.4 and 2.3% improvement than those of a single-head attention-based LSTM model on the DAIC-WOZ and MODMA corpora, respectively. This phenomenon proves that linear projections have a significant influence on the attention mechanism. Linearly projecting the LSTM output into different subspaces and then computing the reduced-dimension context vectors of various subspaces provides more key information than single-head attention.

Table 3 exhibits the results of LSTM and the proposed model. Besides, we also make comparisons with other models mentioned above (Li et al., 2019b; Zhao et al., 2019). We follow the model structure and keep all layer parameters the same to reimplement the models for depression detection. Audios are processed into spectrogram as input features. We use precision, recall, and F1 score as standards for comparison. TP represents the correctly classified number of samples for positive cases. *FP* represents the incorrectly classified number of samples that are misclassified as positive cases and *FN* represents the incorrectly classified number of samples that are misclassified as negative. The calculation of precision and recall is defined as: precision=*TP*/(*TP*+*FP*), recall=*TP*/(*TP*+*FN*), F1=2(precision × recall)/(precision+recall). We use the F1 score as the harmonic mean of precision and recall. The proposed models exceed the LSTM model and the deeper models, 2-D CNN LSTM (Zhao et al., 2019) and CNN LSTM with self-attention mechanism (Li et al., 2019b), in all standards. For the DAIC-WOZ, database, the LSTM+4T model achieves the best F1 score of 0.931 while the LSTM and LSTM+T only achieve 0.907 and 0.915, respectively. For the MODMA database, the LSTM+2T model shows the best performance. It has improvements of 4.7 and 19.4% on precision and recall, respectively, in comparison with those of the LSTM model. The F1 score also increases from 0.859 to 0.987, which indicates the proposed model is effective for depression prediction. Based on the experimental results on the DAIC-WOZ and MODMA corpora, the proposed strategy shows a significant impact on depression detection.

**Table 3:** Comparison between long short-term memory (LSTM) and the proposed model.

	**DAIC-WOZ**			**MODMA**		
**Model**	**Precision(%)**	**Recall(%)**	**F1 score**	**Precision**	**Recall**	**F1 score**
LSTM	89.7	91.6	0.907	94.6	78.7	0.859
LSTM+T	91.6	91.4	0.915	95.5	96.8	0.962
LSTM+2T	91.2	**93.8**	0.925	**99.3**	98.1	**0.987**
LSTM+4T	92.4	**93.8**	**0.931**	96.9	**99.4**	0.981
LSTM+8T	**93.0**	90.8	0.919	98.1	97.4	0.977
Zhao et al. (2019)	91.2	92.0	0.916	92.9	93.5	0.932
Li et al. (2019b)	82.2	89.1	0.855	93.5	90.1	0.918

*Bold values represent the best results in the comparison*.

## 7. Conclusion

In this research, an improved attention-based LSTM network is proposed for depression detection. We first study the speech features for depression detection on the DAIC-WOZ and MODMA corpora. By applying the multi-head time-dimension attention weighting, the proposed model emphasizes the key temporal information. We evaluate the proposed model on both DAIC-WOZ and MODMA corpora. It achieves great superiority over other models for depression classification.

In further directions, first, we may explore other effective speech features for depression detection. Moreover, experiments will be conducted in the future to indicate the efficiency of the modified LSTM model for other time-series predictions.

## Data Availability Statement

Publicly available datasets were analyzed in this study. This data can be found here: The datasets (DAIC-WOZ and MODMA) for this study can be found here: https://dcapswoz.ict.usc.edu/; http://modma.lzu.edu.cn/data/index.

## Author Contributions

YZ designed the overall architecture and wrote the manuscript. ZL conducted the experiments to evaluate the performance of the proposed method. CL and LZ critically refined the manuscript. All authors have contributed to the summary of the presented results and the discussion points and contributed to the review and editing of the manuscript.

## Conflict of Interest

The authors declare that the research was conducted in the absence of any commercial or financial relationships that could be construed as a potential conflict of interest.

## Publisher's Note

All claims expressed in this article are solely those of the authors and do not necessarily represent those of their affiliated organizations, or those of the publisher, the editors and the reviewers. Any product that may be evaluated in this article, or claim that may be made by its manufacturer, is not guaranteed or endorsed by the publisher.
